# Sustainability in a Digital Age as a Trigger for Organizational Development in Education

**DOI:** 10.1007/978-3-030-55878-9_11

**Published:** 2020-07-15

**Authors:** Nina Grünberger, Petra Szucsich

**Affiliations:** 1 Learning, Design and Technology / UNESCO Deputy Chair of Data Science in Higher Education Learning and Teaching, University of Mannheim / Curtin University, Mannheim / Perth, Germany; 2 Department of Education and Social Sciences, University of Cologne, Köln, Germany; 3 Learning, Design & Technology, University of Mannheim, Mannheim, Germany; 4 Human Sciences Faculty, University of Cologne, Cologne, Germany; University College of Teacher Education Vienna, Vienna, Austria

**Keywords:** Digitization, Sustainability, Organization development, Participatory, Wicked problem

## Abstract

As we all know the terms sustainability and digitization are often used these days to describe current social challenges from a global perspective. In order to face these challenges, it is clear that we need an efficient and future-oriented education system. The question arises if the challenges in relation to climate change and digitization change these (learning) organizations as well? And, if yes, in what way? As this chapter points out, sustainability can be regarded as a trigger for an extensive organizational development focusing on ecological, economic (e.g. regarding learning outcomes and assessment) and social aspects. As a consequence, the social structure of the various actors in educational organizations as well as the way in which they interact with each other may/will change as well. In addition to that, climate change and digitization are both so-called *wicked problems* as there is neither a single solution nor a stringent strategy on how to cope with them.

## Introduction

The term *digitization* is originally used to describe the conversion of an analog into a digital system. The origin of *digital* is the Latin word *digitus* for *finger* and especially a *calculating finger* (Han 2013)*.* However, within a post-structural perspective, digitization includes much more than that: Digital technologies have become major instruments for and in our lives. The logic of the binary code is determining social, technical and organizational systems. The architecture of algorithms, codes and data structures is embedded in materiality and infrastructure of our day-to-day life, provides space for communication, articulation, creativity and networking and, therefore, changes self-determination and subjectivization (Jörissen and Verständig 2017, p. 37). According to this, digitization designates a transformational process of social, socio-cultural, economic (Petry 2019) and – we would as well add – ecological structures from both, a local and global perspective.^1^

In the digital age, organizations are, amongst other factors, to a great extent also determined by digitality. New structures, new infrastructures, new jobs and job descriptions as well as new forms of collaboration within educational organizations and between educational organizations with other institutions are needed. In short, the digital age makes a fundamental process of organization development in the educational context necessary (see, e.g., Eickelmann 2010; Grünberger and Münte-Goussar 2017; Schiefner-Rohs 2017; Tulodziecki et al. 2018).

Following a holistic perspective, we can see that digitization affects our living environment much more than previously anticipated. What we have to take into account are the connections “of media technologies, their materiality, hardware and energy, with the geophysical nature” (Parikka 2015, p. 8). Developing, producing, using and disposing of digital media has an ecological, economic and social impact on Planet Earth and our society. Digitization shapes our landscape, our environment and social relationships; sometimes, much more than we could ever have imagined.

As a development parallel to the digital age, individual and political efforts of climate and environmental protection are growing. According to that, educational concepts such as *Education for Sustainability (EfS)* or *Education for Sustainable Development (ESD)* are getting more attention (Chang et al. 2020, p. 1). This might be the result of, on the one hand, political efforts, especially by the United Nations (2015) and their formulation of “Sustainable Development Goals” (short: SDGs) and, on the other hand, the enormous efforts of social movements. Often these social movements grow from a common concern shared by several people into a global movement, like, for instance the Fridays for Future initiative. This means that we can already see some slight changes in the political agenda and efforts towards a sustainable approach, such as “The Green European Deal” by the European Commission (2019).

Similar to *digitization*, *sustainability* is a rather vague term: on the one hand, it describes the maintenance of good conditions for “environmental, economic and social well-being for current and future generations”; on the other hand, it focuses on ensuring the existence on our planet, which means not to “damage” or not to “be harmful to the environment” (Chang et al. 2020, p. 1).

Discussions that connect concerns of sustainability with findings of media (education) studies are rare although they have several things in common (e.g. Clausen et al. 2019; Umweltbundesamt Deutschland 2019; WBGU 2019). They both share a strong focus on research-oriented learning processes and, secondly, both discuss a remarkable change of social structures, for example relationships between teachers and learners in educational organizations. As an approach to take both developments into account, this chapter will discuss structural changes in educational organizations, with a special focus on social relationships and networks. It will point out what it takes to start a participatory approach in organizations and institutions that, consequently, considers both, aspects of sustainability as well as the effects of digitization on our society, on our economy and our ecological environment. In an approach like this, learners and teachers co-create visions of the future and take joint action to start initiatives which are both problem-solving and sustainable in order to handle the big challenges of a digital age during a crucial time in the fight against climate change.^2^

This chapter focuses on the interrelatedness between digitization and efforts concerning a sustainable behaviour for a better future, especially in regard to climate change issues. In the first chapter, some theoretical concepts and empirical research results are explained. Furthermore, we discuss the peculiarity of digitization as well sustainability as so-called *wicked problems* and *crisis-like situations*. From this, we derive implications for transformational processes of educational organizations by becoming more *digital* and more *sustainable*. We finally conclude with some indications for further research in the fields of transformational processes of educational organizations, education for sustainability as well as education in consideration of digitization.

## Sustainability in a Digital Age

Sustainability is a frequently used term, especially in recent years, triggered, for example, by the activities of the Friday for Future initiative and ongoing debates on climate change. From a political perspective, the effort of the United Nations (2015) concerning the SDGs sets an operational framework for further political decisions. Taking into account that the ongoing climate crisis asks for a fundamental change and therefore rapid actions, the European Commission (2019) formulated “The Green European Deal”. In Austria, for example, environmental and climate protection plays an important role in the current policy of the Austrian Federal Government (Österreichische Bundesregierung 2020).

In its origin, the term *sustainability* comes from *to sustain* which means *to bear* or *to suffer* as well as *to keep up* (Harper 2001a). This explanation of the term’s origin already points out the two poles of, on the one hand, striving not to destroy our planet and, on the other hand, trying to preserve the beauty and the balance of our natural habitat for future generations (Harper 2001b). Furthermore, sustainability always includes a social, economic and ecological perspective and their interconnections with each other. At the moment, we can detect a lot of effort concerning environmental and especially climate protection. As Jonathan Franzen (2020) puts it a bit cynically: Maybe we should not concentrate that much on climate protection as we have already lost this fight. He advocates focusing on the giant negative effects of climate change such as droughts and heat, famine, even more exploitation of human beings and our Planet Earth as well as a giant refugee crisis. According to him, all efforts of a so-called green new deal (e.g. Naomi Klein 2019) or a green deal, for instance, the one proposed by the European Commission (2019), are useless and just wasted time and effort. From his perspective, we can see that a unidirectional focus on climate protection and climate change is problematic. As mentioned above, we always have to consider the three aspects of sustainability: ecological, economic and social aspects by regarding past, present and future developments.

Apart from the argument that we are fighting in an already lost “war,” there is another challenge: The trend of “going green” has become popular as people take steps in order to help preserve the Earth for future generations. The development of neo-ecology also concerns ecological, economic as well as societal commitment. It changes markets slowly but noticeably, not only for companies but for consumers as well, as an increasing number of people want to consume in a fair and conscious way. For companies and institutions “going green” is necessary to stay attractive to customers. However, the label “green” does not necessarily mean that the company is really concerned about the environment and acts in an eco-friendly way, as real sustainability may not make business sense. Another example of a development that strives to combine the two aspects of digitization and sustainability is “Green IT,” the practice of using computers and ICT resources in a more efficient and environmentally responsible way. Green IT is “a collection of strategic and tactical initiatives which either: (1) directly reduce the ‘carbon footprint’ of the organisation’s computing operation; (2) use the services of IT to help reduce the organisation’s overall carbon footprint; (3) incentivise and support greener behaviour by the organisation’s employees, customers and suppliers; (4) ensure the sustainability of the resources used by IT” (Hird 2010, p. 16). Green IT could, for example, consider the reduction of the server performance at night or at weekends to reduce power consumption. Research has shown that algorithms, which adjust the performance of a wireless network according to utilization, could achieve an energy reduction of 15% on average (Umweltbundesamt Deutschland 2019, p. 19).

According to Schratz and Steiner-Löffler (1999), we need educational institutions in the digital age that are less concerned about learning issues (reproduction of knowledge) than about life issues (transformation of knowledge). Unfortunately, these questions are often regarded as an irritation rather than a challenge or as something that is not mentioned in the curriculum and therefore not covered at all.

“Going green” in the context of an educational institution could include using recycled paper or reducing the amount of paper used in the first place. At scientific conferences, for example, it has become popular not to print the programme and other print media for sustainability reasons. However, current discussions revolve around the world’s digital carbon footprint, emphasizing the constantly increasing greenhouse gas emissions caused by the transmission of data via the internet and the consumption of electricity by using digital media. This is because the process of transmitting or streaming data requires millions of physical servers in data centres around the world, all spending a lot of energy. Concepts for a more sustainable use of information and communications technology (ICT) often recommend a *reduction* of something: we should reduce the amount of transmitted data, the power consumption, the consumption of streaming services such as Netflix or Spotify, the numerous replacements of digital devices, etc. It is clear that these concepts depend on the social acceptance of an over-all reduction.

However, another example shows the ambivalence and enormous complexity of the topic: Digital technology can also be used *for* climate protection. Some extrapolations point out that ICT tools may even reduce CO2 emissions by up to 20% by the year 2030 (Clausen et al. 2019, p. 1). As one example, the block chain technology is being discussed as a possible means of reducing the emissions of CO2. Again, a lot of parameters need to be considered if you want to decide whether an ICT tool is *sustainable* or not. For instance video-conferencing tools are often referred to as a more sustainable alternative to business trips (especially when traveling by plane). However, research results have revealed that video-conferencing tools reduce business trips just at first sight. On second glance, it has to be taken into account that fewer business trips result in more free time for new projects and new invitations for meetings and, eventually, lead to new occasions for business trips and other forms of required energy (e.g. electricity, amounts of data saved on servers, paper) (Clausen et al. 2019).

*Digital technology* *might also be a means of enhancing environmental und climate protection*. On the one hand, digital technology is used for collecting climate relevant data from around the world. Consequently, digital technology is a main tool for understanding climate change and for monitoring its development (Umweltbundesamt Deutschland 2019, p. 49f). Or, as Chun (2015) puts it: Our idea of climate change is calculated and illustrated by algorithms. Computers collect data, put them in correlation and point out trends of climate change. But they are and always will be calculations and hypotheses and not a blueprint of reality (Chun 2015, p. 678f). On the other hand, digital media and digital visualizations help to make something abstract like climate, climate change and global developments visible and tangible for researchers and citizens. Therefore, we could say that digitization helps us to gather better and more information and awareness about climate change on a local and global scale. Consequently, we can rethink our behaviour and strive to live a more sustainable life (Umweltbundesamt Deutschland 2019, p. 50).

However, in contrast to all aspects mentioned above, we must not forget that *digital technology itself represents an ecological, economic and social challenge* at all stages in the life cycle of digital media: from technology development, the production process, from transport up to the use of digital media and their disposal and/or up- or recycling. In short, digital technology poses a problem for climate protection. In 2019, around 80% of people living in developed countries had a smartphone and used it almost every day. However, a large number of cell phones “can only communicate via networks based on 2G technology, which does not allow using the Internet” (The Shift Project 2019, p. 42). These cell phones are going to be replaced soon. And that is a lot of devices with a lot of energy used for their production and raw materials needed for developing, producing, using and disposing of them. Furthermore, “the number of smartphones will rise from 1.7 billion in 2013 to 5.8 billion in 2020, with a growth of 11% a year” (The Shift Project 2019, p. 34). These arguments concern both, the ecological and economic perspective, but we also have to consider the social perspective: Digital media, which are, for example, used in western countries are produced in China, the raw materials are mostly mined in African countries, where people have to cope with exploitation, child labour and human trafficking. These are practical thoughts concerning the “real” world: this is the “blueprint of reality” mentioned above. We have to think about post- and neo-colonial exploitations (e.g. Castro Varela and Dhawan 2005; Thiébaud et al. 2018) by developed countries and eventually accept our responsibility.

According to this, we can see similarities when we consider the development of the internet: The internet was originally built as a power-free space with equal access for all users (Jörissen and Verständig 2017). However, nowadays, it is far from that. Large corporations like Google, Amazon or Alibaba have the power over the internet. Thinking of other countries from the global south, we have to take into account that internet access is a question of infrastructure like computer hardware, software and wireless network as well as a question of language as most of the information shared on the internet is in English, followed by Russian and German. Smaller languages and minorities have problems to be represented on the World Wide Web. Therefore, standards to represent these minorities are urgently required (Norbert Klein 2018). Still, we can see a huge social gap offline as well as online.

As mentioned above, Jonathan Franzen (2020) emphasizes that we should not focus on climate protection that much, as we have lost this war already. Therefore, Jesse Ribot suggests focusing much more on “climate-related crises”:

“I am definitely not writing about the causes of climate change. I am not writing about smokestacks or drivers in New Jersey or Beijing or anything like that. Rather, I mean the causes of the crises themselves. The causes of hunger, famine, dislocation, economic loss; that is, the outcomes that happen when climate trends or events hit the ground.” (Ribot 2019, p. 34)

As Ribot points out, these climate-related challenges affect “vulnerability,” which is closely related to crises, because “without vulnerability there is no crisis [and …] vulnerability here is the predisposition, in some way or another, to damage”. When vulnerability comes together with hazard and with specific moments like a climate situation, it can easily turn into a social crisis. But: “[…] climate-related crises therefore do not merely fall from the sky when there is a climate event. They are socially produced via conditions on the ground” (Ribot 2019, p. 34).

As sustainability is a far-reaching concept, so is digitization. However, the social transformation triggered by digitization is not only a “more of something.” For example digital technology in everyday life does not only mean reading and writing with the help of digital media. It is a transformation which is changing social structures. Furthermore, digital technology is not only black and white. According to Kerres (2018), it should not be regarded additively, as an additional aspect to our lives but as an integral part. Taking matters a step further, as digital technology makes up an integral part of our lives, thinking and behaving in a “sustainable” and environmentally responsible way should be integral, too. Both, building holistic structures to cope with digital technology *and* behaving sustainably in organizations such as schools and other educational institutions, ask for a fundamental transformation process. Society expects citizens to be able to deal with change constructively, both privately and in the dynamic context of global, multicultural change. According to Fullan, educational organizations are the only social institutions that have the potential to make a significant contribution to this goal (Fullan 1999). However, a transformational process like that leads to questioning well-known, traditional structures and values. Apart from that, speaking of fundamental changes, we often use the term “crises”.

## About Crises and Wicked Problems

One peculiarity of digitization as well sustainability is that they are difficult to define and are often regarded in the context of crisis-like situations. As a modern society, we currently seem to face a lot of *crises*. But crises are not necessarily negative. They are *conditio sine qua non* and *stimuli sui generis* of learning processes and of pedagogy as a research discipline (Schneider-Taylor 2009, p. 104). The etymological origin of the word *crisis* reaches back to the sixteenth century. The Greek word *krísís* literally means separation or decision. A crisis certainly is a kind of turning point (Kluge 2011, Abschn. Krise; Schneider-Taylor 2009, p. 109f). As Koselleck (1973, p. 141) points out, the word crisis was first commonly used in medicine as a decision point between life and death. Therefore, a crisis is often related to the fear of death. In addition to that, the word crisis is closely related to the word *criticism*, which asks for proof and valuation of an issue and can thus be the starting point of a crisis (O’Mahony 2014, S. 250; Pfeifer 1989, p. 934f).

As mentioned above, in order to overcome a crisis*,* traditional structures are being questioned. Additionally, as part of the crisis, socially accepted systems of norms and values have to change as well. After a while, step by step, a new system of structures and a new system of norms and values will be constructed. Another important issue is that with the developments of digitization *and* climate change, we do not only face crises, but so-called *wicked problems*, with challenges which reflect the structural interrelatedness of media, digitization, economy, mankind, ecology and habitat. As stated above, wicked problems cannot “be clearly defined with proposed and testable possible solutions”. They have no “definitive formulation,” “there is no way of determining when a solution has been found; solutions are not true or false but rather good or bad” and “there is no immediate or ultimate test of a solution because any possible solution modifies and changes the problem”. Therefore, wicked problems cannot be solved. The aim must be to understand a wicked problem more and more, to raise awareness and “to learn how to live with it” (Peters 2018, p. 429).

When talking about sustainability and climate change, the term “wicked problem” is commonly used (e.g. Peters 2018). Considering the discourse of digitization, this is, however, not the case. This may seem strange as the speed of the digital development makes it simply impossible to keep up with concepts about possible implications digital technology may have on humans, animals and our Planet Earth, in general. When considering both aspects, we could even speak of “wicked and interwoven challenges.” These challenges have in common that the conditions and many things around them are constantly changing, while people are struggling to solve them. This has various consequences, for instance consequences for social structures: First of all, we have to accept the fact that we do not know and are not able to anticipate how things might develop. Therefore, some projects are open-ended, which means that we may find answers at all levels which will then just lead to even more questions than before. However, crisis-like situations can also be regarded as opportunities for structural changes, for example changes in power structures, and for a more collaborative process of research. It is obvious that this approach cannot follow just one perspective. It clearly requires a holistic, interdisciplinary approach. In addition to that, it has to consider future changes in the short and long run. And, at the same time, it is obvious that this might somehow seem uncomfortable and awkward as uncertainty usually implicates fear and frustration.

## Transformations of Educational Organizations?

How will these “wicked and interwoven challenges” mentioned before transform whole organizations, especially schools and other educational institutions? As stated above, neo-ecology is a trend that will shape the 2020s, as environmental awareness is something companies as well as consumers simply cannot ignore any more. Buzzwords and phrases like energy efficiency, clean energy, greenwashing, “going green,” “Green your product!” are widely used and environmental awareness has become a social movement. As we know, this could be a political or marketing strategy and/or real engagement for climate and environmental protection. In addition to that, we also know that “going green” mainly focuses on ecological aspects and does not primarily consider equal rights and social fairness on a local and global scale. The economic perspective is still the predominant one in our neo-liberal world as it is getting more and more detached from social and ecological aspects. However, as previously mentioned, the consistent reference of ecology, economy and social justice and the relationship among them is urgently necessary, not only in the discourse of digitization.

In the previous chapters, we have already discussed some political initiatives concerning digitization *and* sustainability. Another example is the DigComp – Concept of the European Commission, which provides a framework for major skills that are important in a digitalized world. This framework is often used as a basis for developing educational programmes. It contains one paragraph saying “4.4 Protecting the environment. To be aware of the environmental impact on digital technologies and their use.” (Carretero et al. 2017, p. 17) Furthermore, the German strategy of the Standing Conference of the Ministers of Education and Cultural Affairs (KMK) called “ Education in the Digital World” claims to protect nature and the environment (“4.4. Natur und Umwelt schützen”) (Deutsche Kultusministerkonferenz 2016). In Austria, a new curriculum was developed and put into practice in 2018 for a new school subject called “ Basic Digital Education” (Digitale Grundbildung) in secondary schools, which can either be taught as an individual subject or be integrated in various already existing subjects. In accordance with this new curriculum, children learn about the dynamics and meaning of values, norms and different interests with regard to the use of digital media in various contexts (economic, religious, political, cultural). Furthermore, they are supposed to know to what extent the use of digital technologies damages the environment or contributes to environmental protection (Bundesgesetzblatt für die Republik Österreich 2018). This means, the ecological aspect is mentioned, if only in one point.

To sum up, we can at least see some effort of awareness raising towards ecological aspects in the educational agenda. In addition to that, the efforts of institutions like the German “Wissenschaftlicher Beirat für Globale Umweltveränderungen” (WBGU 2018, 2019) and more or less private institutions like the “Rat für digitale Ökologie” (https://ratfuerdigitaleoekologie.org/) have to be mentioned. The global perspective is taken into consideration, for example, by the United Nation’s (2015) formulation of the SDGs: The SDG 9 targets to “build resilient infrastructure, promote inclusive and sustainable industrialization and foster innovation.” The sub-goal (9.c) aims to “significantly increase access to information and communications technology and strive to provide universal and affordable access to the Internet in least developed countries by 2020.” As a first result, the UN postulated for 2019 “almost all people around the world now live within range of a mobile-cellular network signal, with 90 per cent living within range of a 3G-quality or higher network […]” (United Nations 2015). However, this can be interpreted differently as well: The formulation of the SDG 9 may be (ab)used by ICT companies from western countries to create new jobs to install their infrastructure to the global South without taking heterogeneous cultures, natural environments and social structures into consideration at all. It is obvious that bringing ICT to “least developed countries” is not a good thing per se but has to follow participative processes by regarding the specific conditions of these countries.

After having considered educational policies and the political effort, the question arises what all that means for educational organizations? From our point of view, the social structures of schools as well as of other educational institutions have to change according to “wicked problems,” which cannot be solved and cause discomfort as well as uncertainty in regard to future developments. One important aspect is that the traditional relationship between teachers and students changes as hierarchies are generally levelled down. A lot of local initiatives have emerged, which influence general structures from bottom up. Another important point is that, especially in educational organizations, learners (e.g. pupils and students) as well as teachers can put governing bodies under pressure and thus convince them to take action. We can also see a lot of citizen projects, which first started out locally and then grew into bigger or even global movements such as the Fridays for Future initiative. Fridays for Future has managed to put pressure on educational policy makers to allow the participation on the Fridays for Future demonstrations and to pay more attention to environmental and climate protection in general. All these initiatives have something in common: They have very flat hierarchies. One possible reason for that could be, that all citizens have to act and think like researchers to solve or to better understand a certain “wicked problem.” As there is not the *one* right answer or the *one* approach to find a solution, we all are unknowing but at least trying and learning and are hopefully doing research.

In the context of flat hierarchies, teachers become more like coaches who support learners in individualized learning scenarios. In this special learning environment, subjects, time and space disperse and new paths for cross-curricular learning open up. Learning is no longer confined to the classroom but takes place in companies, on excursions, in museums or exhibitions. The learning time is no longer rigorously squeezed into a 45- or 50-min. cycle, because cross-curricular projects allow students to work on their topic for a longer time and take their own breaks sensibly.

Apart from that, a “wicked situation” like the ones mentioned above requires new forms of research for new solutions in a collaborative and interdisciplinary way. We need to use all our knowledge, imagination, creativity and technology to strive to solve the big challenges of our society. Thus, schools and other educational institutions should allow employees and learners to come together. They should grant them time and space to think about current issues critically and creatively. As a consequence, so-called communities of practice can emerge that share a concern or passion for something they do and, as they interact regularly, they learn how to do it even better and more effectively (Wenger 1998). Professional practice is based on the capacity to reflect on things as one step in the circle of continuous learning. Teachers are experts in many fields like the development of innovative learning designs or the integration of ICT in class. Much of this knowledge, however, is implicit. To share, discuss and reflect on this tacit knowledge with others, teachers often need support to make their competencies visible to themselves and others. In communities of practice, often supported by the supervision and counseling of a university or college, teachers become aware of their competencies and can thus learn and profit from each other’s experience-based knowledge.

In this context, *learning* means learning how to understand the complex developments of our present and future society and trying to find solutions. This approach is not new, as it was already used by the educationalist Wolfgang Klafki (2007). Klafki wrote a didactic concept that describes ways to cope with so-called archetypal, revolutionary key issues (‘epochaltypischen Schlüsselproblemen’). According to him, learning does not aim at developing a verifiable growth of competencies. Consequently, this understanding of learning processes requires open curricula and alternative forms of assessments. In addition to that, this approach is very practice-oriented: After having found a possible solution to a complex problem, this knowledge must be put into action. Without action, the best solution is redundant. But, again, these processes should not happen within a traditional hierarchical structure. All participants of an educational organization – schools and other educational institutions can be regarded as learning organizations in this context – should have the possibility to co-create the organization with the board committees as well as to co-create visions of possible problem-solving strategies and a sustainable future.

Another aspect, which is being discussed in several approaches of “global citizenship education”, “service learning” or “civic education” (Schlicht and Slepcevic-Zach 2016; Sporer and Bremer 2016, p. 356), is a multiple perspectives approach. It is important for educational institutions to start projects cooperating with non-educational partners in order to get a wider perspective and see the big picture. On the one hand, this can be accomplished on a local scale: Local institutions and communities often have needs in specific areas, which may be solved or at least worked on by teachers and learners in cooperation with the local groups. On the other hand, schools profit a lot from external experts who produce new ideas and can enhance the collective understanding of various issues. On a global scale, cooperation or networking with other people, maybe even from different continents, can initiate discussions, raise attention for social inequality and can thus promote tolerance and problem-solving skills.

To sum up, being aware of the transformational power of digital technology, on the one hand, and, on the other hand, of the necessity to cope with environmental and climate protection by acting in a sustainable and environmentally responsible way at all levels – ecologically, economically and socially – can be a trigger for fundamental transformational changes of educational organizations. These changes concern traditional educational structures as teacher-student relationships, time, space as well as the curricula and teaching and learning processes. Becoming aware of these “wicked problems” may consequently also have an impact on individual people and other (learning) organizations on a local and global scale and – in the long run – on national and international political agendas as well.

## Conclusion

In this chapter, we discussed the significance of digitization for climate change as well as a possible means against the further destruction of the environment. Digitization and the idea of sustainability have in common that they can both be seen as “wicked problems” and crisis situations. Thus, both can also be regarded as opportunities and triggers for a necessary transformation of learning organizations to be prepared for future times and for main challenges we, as a society, have to face.

Without any doubt, digitization represents a comprehensive challenge for today’s society. And without a doubt, environmental and climate protection is a very complex issue on a national and international level this century and beyond. But, instead of discussing and meeting these challenges separately, we should deal with them in an interdisciplinary approach, which allows people and institutions to undergo a transformational process that enables them to live and act according to the premises of sustainability. In this context, sustainability has to be understood within the triad of ecological, economic and social aspects. On behalf of this mindset, educational organizations are required to rethink their traditional social structures, economic outcomes and ecological behaviour. As this chapter has shown, sustainability and environmental responsibility include extensive transformational changes in educational organizations and transformational learning processes of all the people involved. Institutions and their people have to think out of the box: Digitization and sustainability are targeting a global perspective, and educational organizations therefore need to open up, reconsider traditional structures and values and include local and global perspectives as well. After all, thinking out of the box may be the one way to face the discussed grand challenges of our society.

In the future, more research has to be carried out in the context of digitization and protecting our environment and climate, in the context of digitization and Education for Sustainability (EfS) as well as digitization and transformations of learning organizations with a specific focus on sustainability. It is obvious that the challenges in the research fields mentioned above can only be met by inter- and transdisciplinary research methodologies.

## Footnotes

1 In the German-speaking discourse, the terms *Mediatisierung* und *Medialisierung* are common (see, e.g., Bettinger and Aßmann 2017). The team *digitization* is rather used as a political and economic term than as a scientific concept.

2 This chapter was basically written in March 2020 during the world-wide crisis triggered by the pandemic of COVID-19. It is clear that this pandemic will have an enormous transformational impact on all organizations, including educational organizations. In many countries, schools are closed, and teaching in schools up to higher education suddenly had to take place in digital form. The restrictions on air traffic, mobility and manufacturing at a global scale may have a positive effect on the fight against climate change. According to these assumptions, it is obvious that the pandemic of COVID-19 will have enormous implications on the topic of this chapter, as it addresses social and organizational transformations by digitization as well as a sustainable approach. However, at the current moment, the consequences of the whole crisis cannot be assessed and in the near future, there will certainly be a lot of research. However, this chapter is not going to include aspects concerning the pandemic as these would just be speculations.
